# Household food (in)security and nutritional status of urban poor children aged 6 to 23 months in Kenya

**DOI:** 10.1186/s12889-015-2403-0

**Published:** 2015-10-13

**Authors:** Maurice Mutisya, Ngianga-bakwin Kandala, Moses Waithanji Ngware, Caroline W. Kabiru

**Affiliations:** African Population and Health Research Center, P.O Box 10787 – 00100, Nairobi, Kenya; University of the Witwatersrand, School of Public Health, 27 St Andrews Road, Johannesburg, Parktown 2193 South Africa; Department of Population Health, Luxembourg Institute of Health, 1A-B, rue Thomas Edison, L-1445 Strassen, Strassen, Luxembourg; Department of Mathematics and Information sciences, Faculty of Engineering and Environment, Northumbria University, Newcastle upon Tyne, UK

**Keywords:** Food (in)security, Urban poor, Child health, Stunting, Malnutrition, Wealth status, Nairobi, Kenya

## Abstract

**Background:**

Millions of people in low and low middle income countries suffer from extreme hunger and malnutrition. Research on the effect of food insecurity on child nutrition is concentrated in high income settings and has produced mixed results. Moreover, the existing evidence on food security and nutrition in children in low and middle income countries is either cross-sectional and/or is based primarily on rural populations. In this paper, we examine the effect of household food security status and its interaction with household wealth status on stunting among children aged between 6 and 23 months in resource-poor urban setting in Kenya.

**Methods:**

We use longitudinal data collected between 2006 and 2012 from two informal settlements in Nairobi, Kenya. Mothers and their new-borns were recruited into the study at birth and followed prospectively. The analytical sample comprised 6858 children from 6552 households. Household food security was measured as a latent variable derived from a set of questions capturing the main domains of access, availability and affordability. A composite measure of wealth was calculated using asset ownership and amenities. Nutritional status was measured using Height-for-Age (HFA) z-scores. Children whose HFA z-scores were below −2 standard deviation were categorized as stunted. We used Cox regression to analyse the data.

**Results:**

The prevalence of stunting was 49 %. The risk of stunting increased by 12 % among children from food insecure households. When the joint effect of food security and wealth status was assessed, the risk of stunting increased significantly by 19 and 22 % among children from moderately food insecure and severely food insecure households and ranked in the middle poor wealth status. Among the poorest and least poor households, food security was not statistically associated with stunting.

**Conclusion:**

Our results shed light on the joint effect of food security and wealth status on stunting. Study findings underscore the need for social protection policies to reduce the high rates of child malnutrition in the urban informal settlements.

## Background

A vast pool of evidence shows that food security is associated with a number of human and economic development outcomes [1, 2]. In light of the high burden of malnutrition and its consequences, the Sustainable Development Goals (SDGs) highlight food security as a human right that needs to be addressed with urgency [3]. Despite this recognition, millions of people in low and low middle income countries suffer from extreme hunger and malnutrition [1]. Household food insecurity is associated with poor nutritional health [2, 4]. Among children, poor nutritional status has negative consequences on growth and development, especially during the early years of life [5]. While poor nutrition status in children includes over nutrition, it often refers to cases of undernutrition [6]. Undernutrition refers to deficiency of protein energy, micronutrients and vitamins as well as minerals essential for growth.

Globally, there has been a decline in malnutrition levels from the 1990s; however, the levels in sub Saharan Africa have remained high [7, 8]. Close to 90 % of stunted (a long term measure of malnutrition) children in the world live in Africa and Asia, with the prevalence of stunting in Africa being 36 % in 2011 [9]. In Kenya the prevalence of stunting was 26 % in 2014 [10]. In urban poor settings in low and middle income countries where few households can produce their own food, the prevalence of stunting may be even higher. In informal settlements in Nairobi, for example, 60 % of children below 5 years were stunted in 2010 as compared to 17 % for the whole of Nairobi [10, 11].

Food security and nutrition are different concepts that are intricately linked resulting to some researchers to apply the two concepts interchangeably. However, food security is a means to nutritional status, and is necessary but not sufficient for nutrition [12]. There exists an extensive body of literature on nutritional status among under-five children and women [1, 13]. The focus on children is because their physiology, growth and development are sensitive to both adequate food (food secure) and nutrition [11, 14, 15]. Further, childhood malnutrition contributes to nearly one third of under-five deaths in the world and up to 11 % of Disability Adjusted Life Years (DALYS), with 80 % of the deaths occurring in low income countries [13].

The first 1000 days of a child life (from conception to the second birthday) have been noted to be very crucial [16, 17]. This period is characterized by rapid brain growth [18]. Therefore, any disruptions, especially occasioned by malnutrition, during this period can have both short and long term effects on education, health and productivity [8].

Research in high income countries shows that food insecurity is associated with poor health outcomes across the population [19, 20]. A study of eight low income countries found food insecurity to be associated with low height-for-age (stunting ) Z-scores [21]. In Canada, food insufficiency was found to be associated with poor self-reported health status [20]. This highlights the importance of food security as a determinant of individual physical, social and mental wellbeing.

Research on the association between food insecurity and malnutrition has produced mixed results. While some studies have found a positive relationship [14, 22, 23] others have found no association or even a negative one [24]. Moreover, the existing evidence on food security and nutrition in children in low and middle income countries is either cross-sectional and/or is based primarily on rural populations. Using longitudinal data collected between 2006 and 2012, we model the relationship between food insecurity and nutrition (measured by stunting) controlling for other covariates among children aged between 6 and 23 months in two urban informal settlements in Nairobi, Kenya. Fotso et al. [11] drew on the same dataset to analyse the effect of poverty on child growth using multi-level modelling. The main outcome was stunting measured by z-scores. Poverty status was measured using several indicators including asset index, household expenditure, food security index and self-reported poverty (based on a subjective index). Overall, the food security index was associated with child stunting for ages 6 to 11 months. The household asset index was also a strong predictor of stunting, and the effect increased with increased age of the child. The study however, modelled the different poverty indicators separately/independently. By so doing, they assumed the different poverty indicators affect stunting in isolation. In addition, the effect of food insecurity on children is hypothesised to take place from the point of introduction to complementary feeding, that is, from 6 months, yet in their study they included all children from birth (age 0). Moreover, as a longitudinal study, which was subject to attrition, it was unusual to use a multi-level model. This model does not take into consideration the loss of follow-up and the time amount of time those lost to follow-up have contributed (exposure duration) to the study, which may have resulted in an underestimate of the effect of the poverty measures on child nutrition.

To address the limitations of the Fotso et al. [11] study, in the current study we used survival analysis, which takes care of the exposure period, hence reducing the bias due to attrition. As food security, a proximate determinant of child nutrition, is likely to be strongly influenced by poverty [13], we interacted the asset wealth index with food security status in order to establish whether the effect of food security differs by the levels of asset wealth status. We therefore hypothesise that 1) household food security is associated with the nutrition status of children aged between 6 and 23 months; and 2) the effect of household food security on stunting does not vary by household wealth status, a proxy measure of poverty.

## Methods

### Study setting

The study was conducted in two informal settlements—Korogocho and Viwandani—located in Nairobi, Kenya. The two study sites are part of the Nairobi Urban Health and Demographic Surveillance System (NUHDSS). The NUHDSS was initiated in 2002 to collect health and demographic statistics from an urban poor population. From 2003, the NUHDSS framework has provided opportunities for nesting studies, including the current study. For detailed description of the NUHDSS see Beguy et al. [25].

### Data and data source

Data for this study come from the Maternal and Child Health (MCH) study (2006–2010), which was a sub-study of the broader Urbanization, Poverty and Health Dynamics project, and the INDEPTH Vaccination Project (IVP) study (2011 – 2013). The latter was a continuation of the MCH study. The MCH study was nested within the NUHDSS framework. The project targeted all women of reproductive health residing in the two study communities who gave birth between the duration of study—2006 to 2013. Under the MCH project, mothers and their new-borns were recruited upon delivery. The mothers and their children were then followed prospectively until the child was 5 years or until when they exited from the study either through death or out migration. Data were collected on the mother’s social demographic characteristics, her health seeking behaviour during and after delivery, feeding practices, immunization of the child as well as the anthropometric measures for both the child and mother. Upon recruitment, three follow-up visits, in this study referred to as updates, were made each calendar year. The following variables were extracted from two data sets: 1) Child characteristics that include date of birth, date of recruitment and subsequent visits, gender of the child, immunization, anthropometric measures, and birth weight and; 2) maternal characteristics that included the mother age at birth, parity, education level and health seeking behaviour. By 2013, 7452 children had been recruited to the study. During analysis, we excluded children with missing information on stunting between the ages 6 and 23 as well as those who were lost to follow-up before they attained the age of 6 months. The final sample consisted of 6858 children contributing to 101,686 person months.

### Variables and measurement

The dependent variable is stunting, which is Height for Age (HFA). Stunting is used here because it is a measure of long term food deprivation (chronic malnutrition) and illness making it a good indicator of child nutrition [26]. We calculated z-scores for the HFA using the ‘*WHO Child Growth Charts and WHO Reference 2007 Charts*’ for children aged up to 2 years. This was suitable because analysis was restricted to children aged between 6 and 24 months. The entry age was set at 6 months, which marks the end of exclusive breastfeeding and introduction to complimentary feeding. The Z-scores show the number of standard deviations of a child on a particular anthropometric measure in relation to a mean or median value. In this regard, those with z-scores of 2 standard deviations of height for age below the WHO reference median were categorized as stunted. Those with a score of above 2 standard deviations were categorized as normal (not stunted) [27]. Child anthropometric measures were obtained during each visit and therefore, stunting was calculated at the each points of visit.

The primary independent variable was household food security status. Food security exists “when all people at all times have access to sufficient, safe, nutritious food to maintain a healthy and active life” [28]. Food security status was computed from a set of questions that captured the domains of food access as described in Radimer framework [29] . The questions assessed the frequency in the 30 days preceding the survey with which households: did not have adequate food; were worried about food availability; lacked enough money to purchase food; and children and adults had to forgo food for a whole day because there was not enough food. The response were coded as either ‘0 = never true’, ‘1 = sometimes true’ and ‘2 = often true.’ The respondent to the food security component was the household head who in his or her absence, the spouse or someone who had enough information and was credible enough was interviewed. Responses were recoded into binary responses: ‘often true’ were coded as ‘1’ and the rest ‘0’ and as described by [30]. We tested for agreement between the items and found a Cronbach’s Alpha of 0.72, indicating a good item reliability [31]. A composite score was generated by summing the items and categorized as 1 = food secure (score of 0); 2 = moderate food insecure (score of 1 or 2); and 3 = severely food insecure (score of more than 2).

The second independent variable was the household asset wealth index, a latent variable computed from a composite measure of household assets and amenities. Principal Component Analysis (PCA) was used to reduce the multidimensional nature of the data to a single score that was categorized into three groups: Poorest, middle poor and least poor [32].

### Statistical analysis

Data were managed and analysed in STATA 13.1. Both descriptive and inferential statistics were used for analysis. Frequencies and percentages were computed to describe the key socio-demographic characteristics of the study sample. In addition, descriptive statistics were used to estimate the prevalence of stunting in the sample. As multiple measures on stunting exist for each child, the exposure, which is the age of the child, was calculated for each visit. We used Cox regression models to estimate the survival time from age six to first stunting and to assess whether the survival time significantly varied by household food security status. The Cox regression models allowed us to control for other known determinants of stunting. In our study we restricted analysis to time to the first stunting. We tested the assumption of proportional hazard in the Cox regression, which is that the hazards are constant between the food security status and wealth status categories being compared. To test this assumption, we used Kaplan-Meier Curves and the log rank test. We also tested the assumption by interacting survival time with time varying covariate. Different Cox regression models were fitted: 1) unadjusted models with the key independent variables that included food security and household; 2) adjusted Cox regression with two main covariates—food security and wealth index; 3) a fully adjusted model, including all the covariates; and 4) a fully adjusted model with all covariates and the interaction between wealth index (poorest, middle poor and least poor) and food security status. The latter model was used to determine whether the effect of food security on stunting was the same across the wealth quintiles.

For the Cox regression model, age in months was the main dependent variable with a dummy variable indicating whether the child is stunted or not. Each child was observed from birth between 2006 and 2013 until either the child was stunted, was censored due to loss of follow-up, out-migration or end of the follow-up for the child who aged above 23 months.

### Ethical clearance and informed consent

Ethical clearance for both the MCH and IVP studies were granted by the Kenya Medical Research Institute (KEMRI). In addition, ethical clearance to use the data for secondary analyses was obtained from the University of the Witwatersrand, Human Research Ethics Committee and AMREF Kenya. Informed consent was obtained from all individual participants included in the study. All procedures were conducted in accordance with the ethical standards of the institutional and/or national research committee and with the 1964 Helsinki declaration and its later amendments or comparable ethical standards.

## Results

Table 1 presents the background characteristics of the study sample at the point of entry into the study (about age of 6 months). The table also shows the proportion of stunted children for each variable category. Overall, the prevalence of stunting among children aged between 6 and 23 months in the study areas was about 49 %. The study sample consisted of nearly an equal number of male and female children, however, the proportion of stunted male children (54 %) was higher than females (44 %). Although a slightly higher proportion of children lived in Korogocho (53 %) than Viwandani, a greater proportion of children in Viwandani (52 %) were stunted compared with Korogocho (45 %).

**Table 1:** Background characteristics of the study sample at entry and levels of stunting (*n* = 6858)

Characteristic	Number		Percentage	
	Total	Stunted	Sample	Stunted
Overall	6858	3349	-	48.83
Child sex^**^				
Male	3462	1858	50.48	53.67
Female	3396	1491	49.52	43.90
Food security^**^				
Food secure	1930	823	28.14	42.64
Moderate	3432	1708	50.04	49.77
Food insecure	1496	818	21.81	54.68
Wealth index^**^				
Poorest	2778	1466	40.51	52.77
Middle	2125	970	30.99	45.65
Least poor	1955	913	28.51	46.70
Mother education^**^				
Incomplete primary/no education	1902	991	27.73	52.10
Completed primary	3251	1621	47.40	49.86
Secondary plus	1705	737	24.86	43.23
Birth weight^**^				
Below 2500	346	166	5.05	47.98
2500 - 2900	1007	548	14.68	54.42
= > 3000	3817	1795	55.66	47.03
No birth weight record	1688	840	24.61	49.76
Breast feeding				
Yes	72	41	1.05	56.94
Introduced to foods	6786	3308	98.95	48.75
Parity^*^				
1	2097	962	30.58	45.88
2	2016	982	29.40	48.71
3	1227	606	17.89	49.39
4	683	346	9.96	50.66
5 plus	835	453	12.18	54.25
Study site^**^				
Korogocho	3613	1894	52.68	52.42
Viwandani	3245	1455	47.32	44.84
HHH Education^**^				
No education	291	151	4.24	51.89
Primary	4172	2117	60.83	50.74
Secondary & above	2393	1081	34.92	45.14
HHH sex				
Female	1606	797	23.42	49.63
Male	5225	2552	76.58	48.59
Mean mother age^a^	6558		25.04 (5.92)	24.73(5.86)
Mean HHH age^a^	6558		34.49 (9.65)	33.92 (9.63)
Average HH size^a^	6558		4.67 (1.98)	4.64 (1.90)

Significant at ^*^ *p* < 0.05; ^**^ *p* < 0.01 when testing difference in the proportion of children stunted by each variables; HH = Household; HHH = HH head; ^a^Mean and standard deviations reported

Two key variables were time variant—food security and household asset wealth index. The two variables were calculated for all the households in the two study sites and thereafter merged with the data used in this analysis. At entry, 50 % of children were from moderately food insecure households, while 22 % were from severely food insecure households. The proportion of stunted children increased with food insecurity. There was a 7 and 12 percentage point increase in stunting between the food secure and either moderate and severely food insecure households respectively. In terms of wealth index, most of the households were ranked poorest at entry time in relation to the overall population in the two study sites. Stunting was highest (53 %) among poorest households and lowest (47 %) among the least poor households.

Mother’s education and parity were significantly associated with stunting. Overall, three quarters of the mothers had at least primary level education. The proportion of children who were stunted was higher (52 %) among mothers with lower levels of education (primary or less) compared with at least secondary education (43 %). Stunting was more common among mothers with a parity of five or more (54 %) compared with those with a parity of below five.

### Food security, wealth status and stunting

Table 2 shows levels of stunting stratified by both household food security and asset wealth status. The results show disproportionate stunting of children across wealth index. The proportion of stunted children was higher among the poorest households than among the least poor. In addition, within each wealth tertile, stunting was higher among children from food insecure households than those from food secure. Stunting was highest among severely food insecure households ranked in the poorest tertile and lowest among food secure households in the middle wealth tertile. These results illustrate the interactive effect of household food security and wealth status on child nutrition.

**Table 2:** Proportion of stunted children by food security status and asset wealth index at the point of stunting

	Number	% of sample	% stunted	*P*-Value
Poorest - overall	2815	41.0	52.50	0.26
Food secure	469	20.2	49.5	
Moderate	1572	94.9	52.5	
Food insecure	774	26.9	54.3	
Middle poor - overall	2072	30.2	46.04	0.01
Food secure	554	23.9	40.8	
Moderate	1009	60.9	46.6	
Food insecure	509	17.7	50.7	
Least poor - overall	1971	28.7	46.52	0.08
Food secure	926	39.9	44.3	
Moderate	859	51.8	47.6	
Food insecure	186	6.5	52.7

**Table 3:** Bivariate and multiple Cox regression hazard ratio on stunting (*n* = 6858)

Variable	M1: Bivariate			M2: Adjusted for WI			M3: Other covariates		
	HR	95 % CI		HR	95 % CI		HR	95 % CI	
Food security									
Food secure	1			1			1		
Moderate	1.16^**^	1.07	1.26	1.15^**^	1.05	1.25	1.12^**^	1.02	1.22
Food insecure	1.23^**^	1.11	1.35	1.21^**^	1.09	1.33	1.15^*^	1.03	1.28
Wealth index									
Poorest	1			1			1		
Middle	0.86^**^	0.79	0.93	0.87^**^	0.80	0.94	0.82^**^	1.02	1.22
Least poor	0.89^**^	0.82	0.96	0.93	0.86	1.02	0.79^*^	1.03	1.28
Study site									
Korogocho	1						1		
Viwandani	0.85^**^	0.79	0.91				0.95	0.86	1.04
Child sex									
Male	1						1		
Female	0.80^**^	0.75	0.85				0.80^**^	0.75	0.86

WI = Wealth index; Model 1 (M1): Bivariate association; Model 2 (M2): Includes both food security and asset wealth index; Model 3 (M3): Includes model 2 and controls for other covariates: Mother and child characteristics (Age at birth, education level, parity at birth, breastfeeding, birth weight) and Household characteristics (head education, sex, age and size)

Significant at ^*^ *p* < 0.05; ^**^ *p* < 0.01

**Fig. 1:**
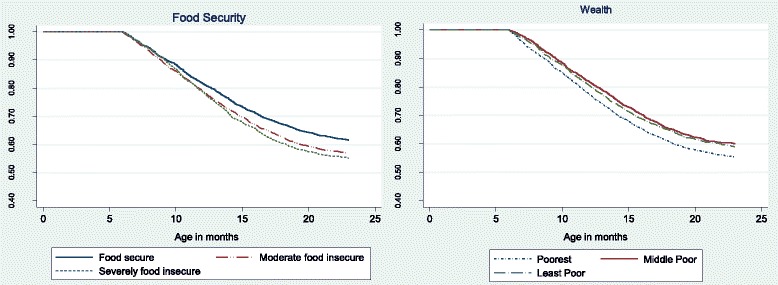
Food security and wealth status Kaplan Meir survival curves

Figure 1 shows the survival curves for both food security and asset wealth index. Children from the poorest and food insecure households had a lower survival time. By 23 months close to half of the children from poor and severely food insecure households were stunted. The food security survival pattern is consistent with descriptive statistics presented in Tables 1 and 2. The wealth index showed no clear difference between the middle poor and the least poor. However, the poorest, had a lower survival than the middle and least poor.

**Fig. 2:**
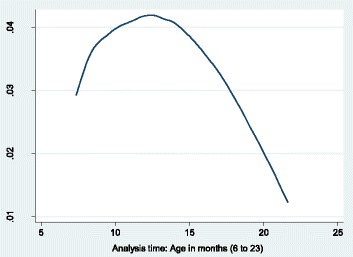
Cox regression survival curve on hazards of stunting

Figure 2 shows the levels of stunting by age of the child in months. Stunting was highest between 10 and 15 months, peaking at around 12 months of age. That is, from 6 months, age at which complimentary feeding is introduced, there was a sharp increase in the proportion of stunted children until 15th month and thereafter declines to slightly about 10 % by month 23.

Using Cox regression models, we further explored the relationship between household food security and stunting (Table 3). Three models are presented: Model 1, with bivariate associations for food security, wealth status, study site and gender of the child; Model 2 includes both food security and wealth index; and a full model (Model 3) which includes Model 2 and controls for other known determinants of stunting. From the bivariate results (Model 1), household security and asset wealth index were significantly associated with stunting. That is, the hazards of a child being stunted if he or she was from a moderate and severely food insecure household increased by 16 % and 23 % compared with children from food secure household. Improved household asset wealth index was associated with lower risk of being stunted. Children from middle (HR: 0.86, CI: 0.79–0.93) and least poor (HR: 0.89, CI: 0.82–0.96) households had significantly lower risk of being stunted compared to those from the poorest households.

After adjusting for the wealth index (M2), the association between food security and stunting remained positive and significant. In this model, children from the severely food insecure households were 21 % (HR: 1.21, CI: 1.09–1.33) more likely to be stunted than those from food secure households. The risk of stunting among moderately food insecure households significantly increased by 15 %; while the effect of wealth index on stunting was attenuated and was only significant for those in the middle category. That is, though the middle poor and least poor had a reduced risk of stunting, only the risk among middle poor remained significant at 95 % level of significance. The third model (M3) included both food security and wealth status controlling for a number of characteristics. Food security in this model remained significant, and was in the same directions as model 1 and 2, while the association between stunting and wealth status was stronger and significant for the middle and least poor category. Other factors significantly associated with stunting in model three were breastfeeding and gender of the child. That is, children who had been introduced to complimentary foods and girls were 37 and 20 % less likely to be stunted compared to those who were breastfeeding and boys respectively.

**Fig. 3:**
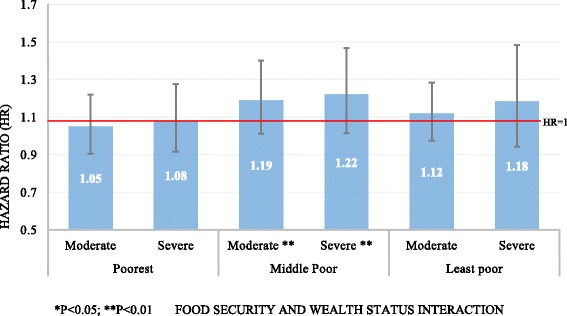
Interacting effect of household food security and wealth index on child stunting

We hypothesized that household food security interacts with asset wealth index to influence stunting. That is, wealth index modifies the effect of food security on stunting. To assess this interaction, we fitted a full model (similar to Model 3) while interacting food security and wealth status (Fig. 3). In the model, we alternated the reference category to food secure households in each wealth status. That is, in the poorest wealth category, the reference group was those households in this category that were food secure, likewise in the least poor wealth category, the reference group was those households in this category that were food secure. In this regard, the same model was run three times with the reference category changing for each wealth category. By so doing, one is able to interpret the hazards ratios within each wealth category more objectively.

Overall, within each wealth category, the risk for stunting increased with increasing food insecurity. However, the association was only significant among the middle poor households. Among the poorest households, the effect of food security on stunting was minimal and not statistically significant. Similarly among the least poor, children from households that were moderate or severely food insecure were 12 and 18 % more likely to be stunted, however, this difference was not statistically significant. Among the middle poor households, moderately and severely food insecure households were at significantly higher risk of being stunted compared with food secure ones.

## Discussion

We sought to determine the relationship between household food security and nutritional outcome of children aged between 6 and 23 months living in two informal settlements in Nairobi using a longitudinal dataset. We hypothesized that food insecurity was a key determinant of stunting and that the effect would be modified by household wealth status. The prevalence of stunting in the study sample was high at 49 % and varied by household food security and wealth status. The prevalence of stunting in this study population was higher compared to the national average of 35 % [33].

The effect of food insecurity after controlling for household wealth status and other known determinants of stunting, showed that children from moderately and severely food insecure households were more likely to be stunted than those from food secure households. Models of food security and child nutrition assume that food insecurity results in a lower intake of energy rich foods and nutrients resulting in changes to child health. A recent study by Ali Naser et al. [34] in Malaysia, showed that food insecurity was associated with stunting and underweight. In contrast, in high income countries the reverse is often true. In high income settings, children from food insecure households have an increased risk of being either overweight or obese than those from rich households presumable due to high consumption of energy rich foods coupled with inadequate physical activity and high stress levels [35]. Closer home, a study among orphaned children aged between 6 and 14 years and living in informal settlements of Nairobi, showed that food security was not associated with their nutritional status [36].

While urban populations are often thought to have better social indicators than rural areas, the growth of slums has eroded this advantage [11]. Research evidence has shown that the urban poor are often worse off than their rural counterparts [37, 38]. Recent research shows that urban poor households are becoming increasingly food insecure, and in Nairobi, levels of food insecurity are estimated to be as high as 85 % [39]. In the slum setting, food insecurity leads to either decreased food intake, or skipping of meals as well as a lack of nutritious foods to meet the dietary needs of household members. The latter is an important composite for growth especially among children. Studies conducted in the two slums have shown that dietary diversification is rare and that many households consume the same foods for most meals [39]. The positive relationship between food security and child nutrition therefore suggests that poor households are not able to meet their daily dietary needs.

When the results were stratified by wealth tertile (poorest, middle poor and least poor), we observed an increased risk of stunting with increasing food insecurity status. However the differences in stunting by food security status were only significant for the middle poor households. The positive effect of the interaction between food security and wealth status is of interest. While many studies have not explored this relationship, the few that have found a diminished effect of food security controlling for wealth index. For instance, a study in Ghana found that controlling for other covariates minus the wealth status food insecurity to be highly associated with stunting of children aged between 24 and 36 months and not those below 24 months [40]. However, when household wealth status (measured in terms of house quality and asset ownership) was controlled for, the effect became insignificant [40]. Hannum et al. [41] in their study on the effect of poverty, food security and nutritional deprivation on literacy achievement found food insecurity to be positively associated with wealth, with the poorest children likely to be both stunted and underweight. In Brazil, Reis [42] investigated food insecurity and its relationship with income and child health and concluded that food security to be a key in influencing the relationship between income and stunting among poor children.

Food security, poverty and nutritional status are closely intertwined, however, their mechanism of transmission remains unclear [40]. While food insecurity leads to decreased dietary intake, poverty is closely linked with increased levels of food insecurity [13]. Poverty, in this regard, modifies the effect of food insecurity on nutrition. In this study, the effect of food insecurity on stunting was only significant among the middle poor households. We think that this significant result is due to the unstable situation of the middle poor category, who are likely to be in a transition phase and can either fall back to the poorest category or move to the least poor. In this regard, households in the middle wealth status may be more diverse. Moreover, with very limited urban agriculture, households in the study setting depend on out-of-pocket food purchases. In this regard, the poverty status may therefore define the household’s access to food. Research evidence on wealth status and obesity shows that in high income countries people from poorer households are more likely to be obese/overweight compared with those from richer households [43]. In high income countries, food insecurity is a measure of poverty, and a strong predictor of overweight, especially among women [44]. In contrast, in low and middle income settings, the poor are less likely to be obese or overweight [43]. Lower levels of obesity and overweight in poorer households in low and middle income countries is often attributed to lower access to energy rich foods and a more physically active lifestyle because the poor have to rely on walking and are more likely to have manual jobs. In the study context, the poorest households irrespective of their food security status, may lack the resources to get enough food for diverse dietary requirements and while the least poor, may have some resources that despite their different food security status guarantees some stability of food. This may be a possible explanation for the lack of statistical significance between the food security categories for the poorest and least poor households.

We interpret the results of this study in light of the following limitations: First, the effect of poverty and food security on nutrition may be more pronounced in older ages than among children. For instance, adult members of the households may miss meals to cope with the existing situations in order to ensure that their children have some food. Saaka et al. [40] found that the relationship between food security and nutritional status was stronger among children aged between 24 and 36 months than those aged between 6 and 23 months. However, the importance of studying those aged between 6 and 23 months is twofold: it is a critical age for growth and in the study context, nearly half of the children are stunted by this age. Secondly, household wealth was assessed using proxy measures—household amenities and asset ownership—due to a lack of accurate information on household incomes and expenditures, which may be better measures of household wealth. Despite these limitations, we utilize longitudinal data from the same children and households providing an opportunity for survival analysis, which is superior to cross-sectional analysis. The longitudinal nature of the data also allowed controlling for fluctuations in household food security and wealth status over time, by modelling the two variables as time variant. By so doing, the probability of reporting a significant effect when there is none was reduced [45].

## Conclusion

The findings of this study show food insecurity is intertwined with malnutrition and poverty. Food insecurity is a public health problem and increasingly becoming a social determinant of health [46, 47]. Given that food security is a human right [47], there is need for social protection policies to reduce the high rates of child malnutrition in the urban informal settlements. Previous efforts by Concern Worldwide in Korogocho slums showed that social protection strategies through cash transfers increased the mean number of meals from 1.6 to 2.5 in a day and decreased prevalence of food insecurity from as high as 97 to 74 % [48]. Further, targeted efforts to address malnutrition among children are imperative. The interventions must take into account high levels of poor sanitation and diarrhoea, which may erode the gains of improved food security among the slums residents [49]. As such, interventions must take a holistic approach in addressing the high levels of malnutrition among the vulnerable urban poor households.

## Abbreviations

AMREF: African Medical and Research Foundation
APHRC: African Population and Health Research Center
DALYS: Disability adjusted life years
HFA: Height-for-age
HR: Hazard ratio
INDEPTH: International Network for the Demographic Evaluation of Populations and Their Health in Developing Countries
IVP: INDEPTH vaccination project
KEMRI: Kenya Medical Research Institute
MCH: Maternal and child health
NUHDSS: Nairobi Urban and Health Demographic Surveillance System
PCA: Principle component analysis
SDGs: Sustainable development goals
WHO: World Health Organization
